# Can all healthy adults use the current evidential breath alcohol analysers? An investigation using a large spirometry database

**DOI:** 10.1177/00258172231178419

**Published:** 2023-06-13

**Authors:** Galen Ives, Laura Sbaffi, Peter A Bath

**Affiliations:** Information School, University of Sheffield, UK

**Keywords:** Breath alcohol, breathalyser, Failure to Provide, Intoxilyzer, spirometry

## Abstract

People failing to give a specimen of breath at a police station are assumed to be deliberately obstructive and are charged with Failure to Provide under the Road Traffic Act 1988. However, spirometry records of 281,210 healthy individuals from UK BioBank showed that a significant minority cannot use existing evidential breath analysis machines. Women were three times more likely to be unable to use them than men (1.64% vs 0.54%) with the risk rising with age six-fold from those in their 40s (0.43%) to 2.7% in their 70s, with women more affected (0.65% to 3.8%). Short stature was a further risk factor: 2.6% of men and 3.8% of women below the 2^nd^ percentile of height could not use the current machines, with almost one in ten elderly, short women unable to do so, while smokers aged 50+ were twice as likely as non-smokers of the same age to be unable to provide breath specimens.

## Introduction

In the UK, three desk-top analysers for breath alcohol currently have had Home Office Type Approval^1^ since 1998; these are the Lion Intoxilyzer 6000, the Intoximeter EC/IR and the Camic Datamaster; the last of these is out of production and used by only four police forces.

On occasion, a person using an evidential machine fails to provide a valid sample whilst maintaining that they have tried their best. This normally leads to a charge of failing to provide a specimen under the Road Traffic Act 1988; there may be a defence if there is a proven history of a condition such as asthma or chronic obstructive pulmonary disease (COPD) and the administration protocol^2^ requires officers to enquire about any such medical conditions.

As medical science has advanced, spirometry has been deployed to validate methods of obtaining breath samples for evidential purposes.^3–8^ Much of this research has investigated impaired individuals and information on healthy individuals' ability to use the currently approved equipment is quite sparse – fewer than 300 healthy individuals, i.e. those without lung disease, have been investigated. This paper aims to improve our understanding in this area using the large UK BioBank database of data from over half a million UK volunteers.

Spirometric assessment provides three principal parameters:

- Peak expiratory flow rate (PEFR): the person blows as hard and fast as possible into a wide tube; the maximum rate of flow is recorded.
- Forced expiratory volume in 1 second (FEV1): the volume of air the person exhales in the first second when exhaling as forcefully as possible.
- Forced vital capacity (FVC): the total volume of air that the person is able to exhale forcibly from one full breath.

### Overview of existing research

Reporting in 1991, Gomm et al.^3^ investigated 51 individuals with various respiratory disorders, of which 29 individuals (57%) were unable to provide satisfactory evidential breath samples using either the Lion 3000 or Camic devices. A further paper by Gomm et al.^4^ in 1993 investigated 48 persons of “small stature”, of whom over a quarter (13 or 27%) could not fulfil all of the requirements of the devices tested.

A research programme set up by Lion Laboratories, the makers of the Intoxilyzer, was reported in 1997 by Williams et al.^5^ testing the new Lion Intoxilyzer 6000. Ninety-seven participants comprised an unstated age cross-section of 40 “normal” participants, 26 “large and fit” rowers, 11 individuals of “small stature” and 20 hospital outpatients with an undefined mix of respiratory disorders of unstated severity. All except two in the outpatient group were able to provide breath samples and the authors concluded that the Lion 6000 was suitable for general use.

The same research group in 2000 (Honeybourne et al.^6^) investigated 40 adults using the Lion Intoxilyzer 6000; the sample comprised 10 each of healthy controls, and people with asthma, COPD and restrictive lung disease. Of these, a total of 9 failed even after 4 attempts; 7 of these had an FEV1 below 1.5 litres, and of the 9 people overall whose FEV1 was below 1.5 litres, 7 failed.

In 2016, Seccombe et al.^7^ investigated 26 people with COPD and 24 with ILD (interstitial lung disease or pulmonary fibrosis), classified as “moderate” or “severe”. The study used the Lion Intoxilyzer 8000 in use in Australia, similar to but more recent than the 6000 model approved for use in the UK. It was found that no individual with an FVC below 1.5 litres was able to use the machine.

A single paper from 2001 by Stephens and Franklin^8^ specifically investigated the level of lung function required to operate the Camic Datamaster. A total sample of 259 comprised 142 participants from the healthy population, 94 from local chest clinics and an additional sample of 23 minors aged 6 to 14 years. Nine participants in total failed to provide a valid sample, all of these being chest clinic patients, but no detailed analysis of the findings was presented beyond three scatterplots. A scatterplot of actual FEV1 vs percentage of predicted FEV1 (i.e. what would be expected for each individual given their age, sex and height) clearly separated those who succeeded from those who failed. All who failed had both an actual FEV1 below 1.5 litres and a predicted FEV1 of 60% or less: combining these two variables correctly identified all 9 of the failures and only one of the 259 who succeeded, this exception being a child of 10 years.

### Implications for current usage

Despite the sparsity of existing research, there are quite clear indications that some healthy individuals may have difficulty using evidential breath machines. An FEV1 below 1.5 litres is strongly associated with failure^6,8^ and the predictive value of this is greatly strengthened when the expected value for a person’s FEV1 is 60% or less.^8^ For the purposes of the present research, a person defined as “low FEV1” meets both of the above criteria.

## Methods

Spirometry data, including FVC and FEV1, was available for 353,284 of the volunteers from the UK BioBank database; all gave their ethnicity as “White” or “Irish”. The final data set comprised 281,210 non-smoking individuals with no reported history of any lung disease or respiratory complaint after the following exclusions:

- Asthma (10.9%; n = 38,458).
- Emphysema or chronic bronchitis (1.3%; n = 4,472).
- Blood clot in the lung (0.5%; n = 1,682).
- Any ICD-10 diagnosis of other respiratory disease (2.0%; n = 6,967).
- Those who reported being daily smokers (7.7%; n = 27,156) were also excluded from the main analysis but examined as a separate group.

## Results

*Note:* Statistical tests of significance are not given here because the very large dataset results in extremely high levels of significance (typically p ≪ 10^−6^) even for small numerical differences. All the findings below are statistically very robust.

**Figure 1.**
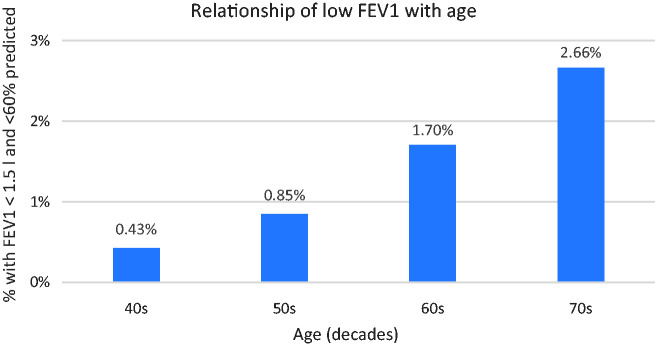
Increasing frequency of low FEV1 in relation to age group.

An initial analysis of the final data set revealed that 3,176 individuals, or 1.13% of the sample, recorded a low FEV1 by the criteria defined above; there was a threefold sex difference, with 0.54% of males and 1.64% of females meeting the low FEV1 criteria, and, as would be expected, respiratory competence declined with age as shown in Figure 1.

**Figure 2.**
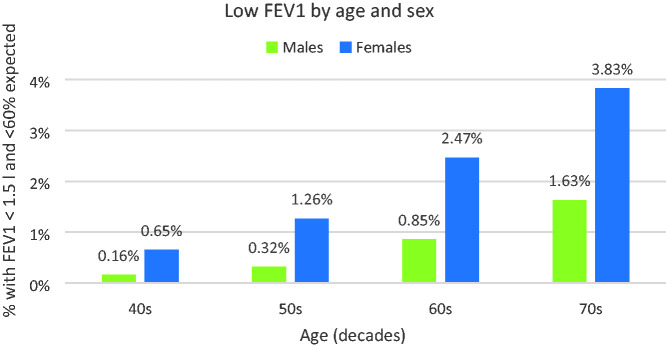
Sex differences in age-related increase in people with low FEV1.

A higher percentage of females have low FEV1 in all age groups, as shown in Figure 2.

### People of small stature

**Figure 3.**
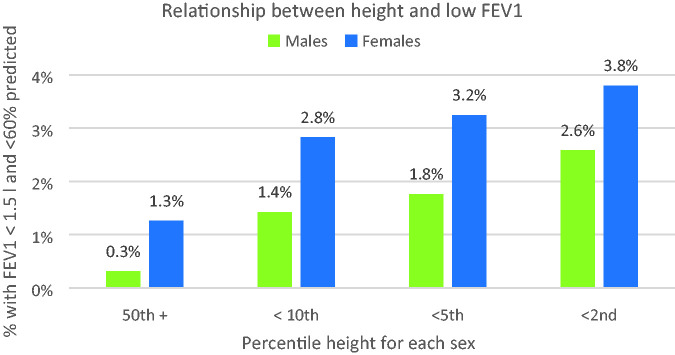
Proportions of low FEV1 at various height percentiles for each sex.

A strong relationship between low FEV1 and height emerged, again with noticeable sex differences, as shown in Figure 3.

**Figure 4.**
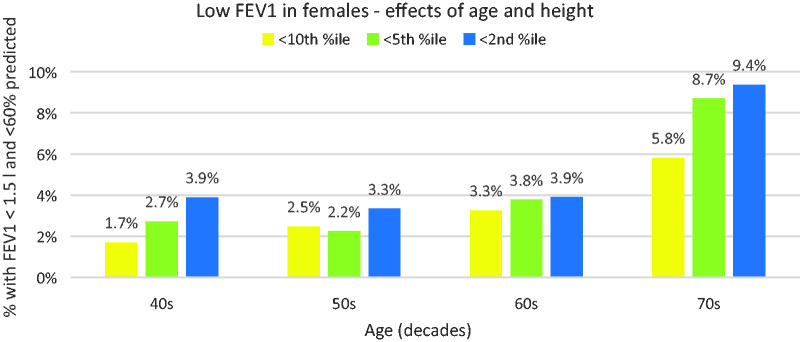
Change in proportions of low FEV1 with age for females of small stature.

As might be expected, age and height interact in their effect on the proportion of subjects with low FEV1; Figure 4 illustrates how females of small stature (tenth percentile and below) are increasingly at greater risk of low FEV1 as they age.

### Smokers

**Figure 5.**
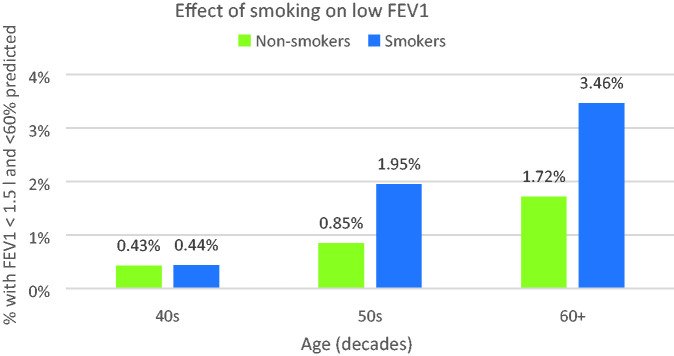
Age-related increase in proportion of smokers with low FEV1.

Daily smokers comprised a relatively small proportion of the overall sample (23,266 individuals or 7.6% of those with no lung disease); more of these had low FEV1 – overall 2.1% compared with 1.1% of non-smokers, and again the difference in risk increased with age; this is illustrated in Figure 5.

**Figure 6.**
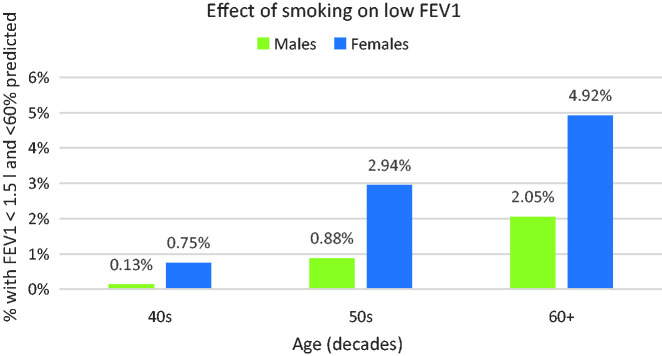
Sex differences in low FEV1 in smokers.

Figure 5 shows that smoking more than doubles the risk of low FEV1 in age groups aged over 40 with females disproportionately worse affected – overall, 1.15% of male and 3.12% of female smokers met the low FEV1 criteria; Figure 6 illustrates this for different age groups:

## Discussion

The general assumption of the police and the courts is that those who fail to provide a breath specimen are wilfully failing to do so but evidence for this assumption is extremely thin. The literature search revealed only four papers pertaining to the current evidential machines and there are difficulties with each of these because of small samples, little detailed analysis or commentary, and risk of bias.

### Effects of stature

It is very clear from the BioBank data that shorter persons are at greater risk of being unable to provide a breath sample:

- Whilst few men (0.3%) of average height or above are at risk, this increases eightfold to 2.6% for those below the 2^nd^ percentile.
- Women of average height and above are already more at risk than similar men at 1.3%, and this increases threefold to 3.8% for those below the 2^nd^ percentile.
- Elderly women (70 and over) are particularly at risk – almost 1 in 10 of the shortest in this age group would be unable to use evidential machines.

### Effects of age

Age is also an important factor:

- Risk approximately doubles with each decade from the 40s to the 60s.
- Comparing the youngest with the oldest (40s vs 70s), the risk increases tenfold for men (0.16% vs 1.63%) and sixfold for women (0.65% to 3.83%).
- As noted above, there is an interaction between age and stature, with short, elderly persons least likely to be able to provide a sample, and this is exacerbated if they are female.

### Effect of sex

The sex of the person clearly emerges as critical factor:

- Overall, nearly four times as many females would be unable to provide an evidential breath sample as males, although this difference decreases with advancing age.
- In comparisons based upon age, stature or smoking status, sex remains an important factor, with women being more at risk than men in all circumstances investigated.

### Effects of smoking

No previous study has investigated the effects of smoking tobacco despite its well-known deleterious effects upon lung function. The present investigation confirms its relevance:

- Smoking approximately doubles the risk of being unable to supply breath samples in those beyond the 40s decade.
- About 1 in 20 female smokers in their 60s would be unable to supply breath samples.

## Conclusions

The spirometric criteria chosen here on the basis of Stephens and Franklin^8^ are quite stringent and the figures given in this paper for those unable to use the extant evidential machines should be considered *minimum* values.

This study implies that, overall, at least 1 man in 186 and 1 woman in 61 would be physiologically incapable of providing an evidential breath sample and these figures can be approximately halved to 1 man in 87 or 1 woman in 32 if they happen to be daily smokers. Age increases risk, with people in their 70 s being six times more likely to fail than those in their 40s. With regard to stature, the risk figures rise to 1 in 38 short men and 1 in 26 short women (i.e. below the second percentile of height), with increasing age further compounding this effect.

There are around 4000 annual prosecutions in the UK for Failure to Provide under the 1988 Act.^9^ If, as our results imply, a percentage of the population are physiologically incapable of operating the extant machines, then some of these annual prosecutions may have had a wrong outcome – some individuals who should have actually received a penalty for driving under the influence of alcohol may have been acquitted when a different specimen would have proved their guilt, whilst other individuals who were not, in fact, over the legal limit may have been wrongly convicted of Failure to Provide simply because they were unable to use the machine. It is not possible to estimate the actual number of unsafe convictions without detailed demographic information (age, sex, height, smoking status) regarding those who were prosecuted.

Correcting this situation would not require legislation but merely alterations to existing procedures, as the 1988 Act allows for a person to give an alternative sample if “the constable who required the specimens of breath has reasonable cause to believe that the device has not produced a reliable indication” or if “it is then for any other reason not practicable to use such a device”. It would be helpful if police forces were alerted to the fact that certain people are unable to use the extant evidential machines and adopt a more flexible approach in allowing an alternative sample to be taken.

## Limitations and further work

This paper has relied on published research which is statistically and methodologically of relatively low quality, for which reason the most stringent criteria were adopted and this may have underestimated the proportion of people who would in fact be unable to use the existing machines. This study was theoretical only, in that spirometric measurements were not tested against actual evidential machines. The BioBank sample comprises exclusively volunteers which may have introduced an unquantifiable bias. The BioBank sample contains spirometric data only for individuals giving their ethnicity as “White”, “White British”, “Irish” or “Other white” and there is therefore no information regarding other ethnic groups.

Useful further work would involve spirometric measurements of a representative sample stratified by age, sex and height, large enough to give adequate statistical power, coupled with tests using evidential breath analysers; we would strongly recommend that manufacturers of such machines undertake and publish such research.
